# Highly Ordered Single Domain *Peri*-Tetracene Monolayers on Ag(110)

**DOI:** 10.1021/acs.jpcc.5c01482

**Published:** 2025-04-15

**Authors:** Maren Zirwick, Nina Kainbacher, John B. Bauer, Marie S. Wagner, Peter Puschnig, Thomas Chassé, Holger F. Bettinger, Heiko Peisert

**Affiliations:** † Institute of Physical and Theoretical Chemistry, University of Tübingen, 72076 Tübingen, Germany; ‡ Institute of Organic Chemistry, University of Tübingen, 72076 Tübingen, Germany; § Institute of Physics, NAWI Graz, University of Graz, 8010 Graz, Austria; ∥ Center for Light-Matter Interaction, Sensors & Analytics (LISA+) at the University of Tübingen, 72076 Tübingen, Germany

## Abstract

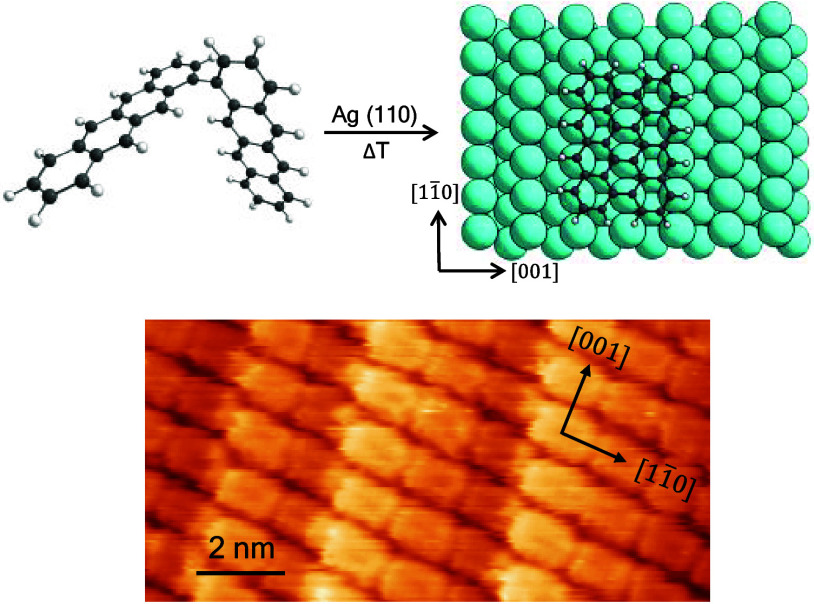

The on-surface reaction of 1,1’-bitetracene (Bi4A) to *peri*-tetracene (tetrabenzo[bc,ef,kl,no]coronene) (4-PA) on Cu(110) and Ag(110) is studied by photoemission, scanning tunneling microscopy (STM) and low energy electron diffraction (LEED). Density functional theory (DFT) computations suggest that the Ag(110) substrate is well suited for the formation of large-area 4-PA monolayers with a preferential adsorption alignment of 4-PA molecules along the [11̅0] direction. The experiments confirm the formation of 4-PA and presence of large highly ordered 4-PA domains. Two distinct phases emerge, growing seamlessly over large areas and even spanning step edges. Evidence for charge transfer from the substrate to the molecule was found, resulting in a filling of the lowest unoccupied molecular orbital (LUMO) of 4-PA.

## 1 Introduction

The “bottom-up” chemical synthesis of nanographenes (NG) with perfectly defined structures and properties represents a significant milestone for applications in the field of nanoelectronics. Nanoscale graphene fragments such as acenes and *peri*-acenes in particular, offer great potential for creating customized bandgaps for organic semiconductor materials. In addition, these materials have attracted great interest due to the ability to control precisely their atomic-scale structure, and their seamless integration into a manifold of electronic devices.^1−7^ The general structure of nanographenes, particularly the width and edge structure, has a significant impact on their electronic, magnetic, and chemical properties.^8−15^ Additionally, the presence of diradical centers plays a crucial role in shaping these properties.^16^

The growth of NG is highly dependent on the type of substrate used.^17^ It was demonstrated that the comprehensive understanding of interactions at interfaces is crucial for the selective synthesis of graphene-based materials.^17^ The choice of the metal substrate influences not only the alignment and orientation of the molecules, but also their electronic and structural properties. Especially domain boundaries are a determining factor in the performance of organic devices.^18^ Metal substrates can provide catalytic support during the growth process, promoting the formation of nanographene fragments with specific characteristics, such as particular edge configurations or chirality.^17,19^ Additionally, the interaction between the nanographene fragments and the metal surface can affect the overall morphology, growth rate, and quality. Variations of the metal surface, such as differences in roughness or crystallographic orientation, can lead to significant differences in the behavior and properties of the grown nanographenes, highlighting the critical role of substrate selection in controlling the synthesis of high-quality NGs.^1,19−22^

*Peri*-acenes, which are composed of linearly fused benzene rings and feature a two-dimensional expansion of the π-conjugated electron system, are ideal model molecules for studying the electronic structure of large zigzag nanographenes.^23−25^ The synthesis of these molecules is challenging due to the highly reactive nature of the molecules. Solution-based synthesis is often limited to the production of *peri*-acene derivatives, with stabilized reactive sites, either kinetically by sterically demanding substituents or thermodynamically by the introduction of heteroatoms.^26−32^*Peri*-acenes can also be synthesized on surfaces through the use of appropriate precursor molecules, which undergo oxidative ring-closure or metal-catalyzed cyclodehydrogenation reactions. This has been demonstrated for 4-PA, 5-PA, and 7-PA, which were synthesized on the Au(111)^33−36^ or Cu(111)^37^ surface from (sub)monolayer films of the respective precursor molecules. While only individual *peri*-acene molecules could be synthesized on the Au (111) surface,^33−36^ smaller areas of highly ordered 4-PA molecules were successfully generated on the Cu(111) surface.^37^ However, the preparation of large, homogeneous, and highly ordered 4-PA regions remains a challenge. In this work, we demonstrate that utilizing the semireactive Ag(110) surface represents a substantial advancement toward achieving this goal.

## 2 Methods

The 1,1’-bitetracene (Bi4A) precursor was synthesized as described previously.^37^ The molecules were evaporated from a Knudsen cell at a rate of 0.1 nm/min, as determined by a quartz crystal microbalance. During the evaporation process, the single crystal was held at room temperature. The evaporated monolayer was then annealed at 250 °C for 30 min to induce cyclodehydrogenation. Film thickness values were obtained by comparing photoemission intensities between the substrate and the overlayer-related peaks. Atomic cross sections were taken from Yeh and Lindau,^38^ while the mean free paths for organic molecules were calculated according to Seah and Dench.^39^ Based on the crystal structure of related pentacene, the molecule-to-molecule distance in vapor-deposited crystals is approximately 0.35 nm.^40^ Assuming the molecules do not lie completely flat, the thickness of a nominal monolayer (ML) was estimated to be 0.4 nm.

Photoemission (PES) measurements were performed on a multichamber ultrahigh-vacuum system equipped with a hemispherical energy analyzer (Phoibos 150, SPECS), an X-ray source (Al Kα radiation, *hν* = 1486.7 eV) with a monochromator (XR 50 M, SPECS), and a UV radiation source (UVS 300, SPECS). For UPS measurements, excitation energies of He I (21.2 eV) and He II (40.8 eV) were used. The photoemission spectra were calibrated by reproducing the binding energies (BE) of Au 4f_7/2_ and Cu 3p_3/2_ at 84.00 and 932.56 eV, respectively. The core-level spectra were fitted using Unifit 2018.^41^ A Shirley model was employed for the background, while a Voigt profile, which combines Lorentzian and Gaussian components, was used for peak fitting. The Lorentzian width for the C 1s core-level was set to 0.10 eV, according to the literature.^42^ The absolute binding energy error is estimated to be within ± 0.05 eV. To account for the asymmetric peak shape of the C 1s core levels, the Doniach–Sunjic peak shape^43^ was applied.

STM and LEED measurements were performed in a two-chamber UHV system equipped with a LEED/AES spectrometer (Auger electron spectroscopy) from OCI Vacuum Microengineering Inc. and a variable temperature (VT) STM from Omicron NanoTechnology GmbH. The base pressure in the measuring chamber was at around p = 5.0–10^–11^ mbar and for the STM measurements, mechanically cut Pt/Ir tips were used. Both the sample and the tip were maintained at room temperature, with all tunneling voltages referenced to the sample. The STM images were processed with the WSxM program.^44^ In some cases, simple smoothing (subtraction of an average value) was applied or only the image contrast and brightness were improved. The LEEDpat program^45^ was used for the LEED analysis.

Geometry optimizations employed the B3LYP^46−48^ functional together with the def2-TZVP basis set^49^ and were performed with Orca.^50^ The on-surface computations were performed using the Amsterdam Modeling Suite (ams).^51^ The DFTB (density functional tight binding) method with the GFN1-xTB method developed by Grimme^52^ was used to compute the preferred adsorption geometry. Three-layer Ag(110), Cu(110), and Cu(111) surfaces (7 × 7 of atoms) were used. The atoms of the two lower layers were fixed, while the atoms of the top layer were relaxed during the computation. A natural population analysis (NPA) was performed with the NBO program.^53^

To get a more accurate description of energetics of the adsorption and the electronic structure of the adsorbate layers, density functional theory (DFT) calculations with VASP^54−56^ have also been conducted. Here, 4-PA was adsorbed on Ag (110) in a  unit cell and Bi4A in a  unit cell, both on 5 layers of Ag(110) keeping the lowest 3 layers fixed during the geometry optimization. The geometry of these two systems was first optimized on the preferred adsorption sites using the GGA-PBE^57^ exchange correlation functional and the DFT-D3-zero method of Grimme^58^ for van der Waals corrections. Here, a 3 × 3 × 1 Mohnkhorst-Pack k-grid^59^ was used. To get a more realistic description of the electronic structure, the more accurate hybrid functional HSE06 has been utilized to obtain the molecular orbital projected density of states (MOPDOS). For the latter single-point calculations, a 2 × 2 × 1 Monkhorst–Pack k-grid has been used.^54^

## 3 Results and Discussion

### 3.1 Choice of the Substrates

The selection of the type and the surface geometry of a substrate plays a critical role for both chemical (surface-)reactions and the adsorption geometry of the molecule. The geometry and type of the metal surface may support the formation of highly ordered monolayer films. Copper substrates are well suited for surface-assisted cyclodehydrogenation reactions. On Cu(111), the formation of *peri*-tetracene (4-PA) from a 1,1’-bitetracene (Bi4A) precursor was observed after annealing to 250 °C.^37^ The 4-PA molecules are oriented along three rotational domains along the [1̅10]-direction of the copper surface which results in an island formation.^37^ Thus, considering the symmetry of a *peri*-tetracene molecule, the 3-fold crystal symmetry of the Cu(111) substrate may hinder the formation of larger 4-PA monolayer films.

The rectangular surface symmetry of Cu(110) might support a more defined orientation of 4-PA molecules. It is known that tetracene (4A) adsorbs preferential with its long axis parallel to the [11̅0] direction of the Cu(110) substrate,^60−62^ as an acene molecule (benzene ring: 2.46 – 2.52 Å)^63^ matches the distance of a copper unit cell along the [11̅0] direction almost perfectly (2.55 Å). However, our experiments show that 4-PA molecules on Cu(110) are oriented in both the [001] and [11̅0] direction of the substrate surface (Figure S1). A possible reason could be the geometry of the precursor molecule Bi4A. DFT calculations show that due to steric hindrance by the H atoms, the “4A wings” of an isolated molecule are not parallel to each other, but rather form a dihedral angle of 107°.^37^ Although a partial planarization of the molecule during adsorption (with one wing lying flat on the surface while the other wing shows some tilting) is observed,^37^ a complete planarization and parallel alignment of the “4A wings” is not possible. Therefore, we assume that on a substrate surface geometry with a perfect fit to the acene structure, the adsorption of Bi4A on the energetically favored adsorption sites results not in the formation of a most densely packed monolayer. For higher coverage, energetically slightly different adsorption sites might be occupied.

In contrast to the Cu(110) surface, the Ag(110) surface exhibits a slightly larger unit cell distance along the [11̅0] direction (2.88 Å) compared to copper and thus offers the opportunity for the Bi4A molecule to twist the flat lying 4A wing. This allows more flexibility of both 4A wings to adsorb on the surface. On the other hand, it can be said that a silver substrate is less reactive than a copper surface,^64,65^ which might affect on-surface reactions.

Therefore, we computed adsorption energies of the molecules on Cu(111), Cu(110), and Ag(110) surfaces (Table 1). Calculations were done for Bi4A and 4-PA lying in [11̅0] or [001], and [11̅0] or [112̅] direction, respectively. The four main adsorption sites were chosen as the initial adsorption sites, namely top, bridge, hollow-hcp and hollow-fcc for the (111) geometry and top, hollow, short bridge and long bridge for the (110) crystal geometry (cf. Figure S2, Supporting Information).

**Table 1:** Computed Adsorption Energies of Bi4A and 4-PA on Different Substrates (GFN1-xTB Method)^a^

	Cu (111)		Cu (110)		Ag (110)	
	Bi4A	4-PA	Bi4A	4-PA	Bi4A	4-PA
[11̅0]	**4.69 eV**	**5.28 eV**	**5.77 eV**	6.33 eV	**3.71 eV**	**4.93 eV**
[112̅]/[001]	4.68 eV	5.23 eV	**5.77 eV**	**6.42 eV**	3.66 eV	4.78 eV
Δ*E*_ads_	*–*0.01 eV	–0.05 eV	±0 eV	+0.09 eV	–0.05 eV	–0.15 eV

a The bold letters highlight the preferred adsorption direction of Bi4A and 4-PA on the different metal substrates.

The results for Bi4A and 4-PA on Cu(111) are in good agreement to recently published computations analyzed by DFT.^37^ The optimal adsorption sites are the hollow-hcp sites along the [11̅0] direction.

The preferred adsorption sites of Bi4A along the [11̅0] and [001] direction on the Cu(110) surface exhibit no difference in their adsorption energy (Table 1). Also, for 4-PA/Cu(110) only a slight difference of 0.09 eV has been computed (Table 1). These computational results are consistent with STM images of annealed Bi4A (= 4-PA) on Cu(110), which show an inhomogeneous layer with adsorbed 4-PA molecules in [11̅0] and [001] direction (Figure S1).

**Figure 1:**
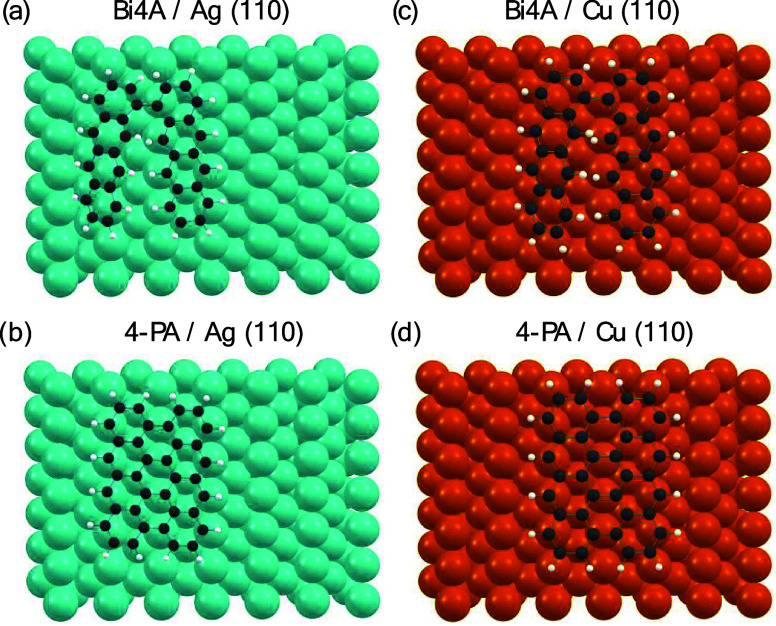
Preferred adsorption site of Bi4A and 4-PA on Ag(110) (a, b) along the [11̅0]-direction and adsorption site of Bi4A and 4-PA on Cu(110) along the [11̅0]-direction (c, d) as computed using the DFTB method.

In contrast, on Ag(110) the adsorption sites for Bi4A and 4-PA along the [11̅0] direction are energetically more clearly preferred (Table 1). These adsorption geometries are illustrated in Figure 1 and Figure S3 (Supporting Information). 4-PA shows a difference in adsorption energy in the [11̅0] direction compared to the [001] direction by 0.15 eV. This may support a homogeneous growth of 4-PA molecules along the [11̅0] direction, which will be investigated experimentally in the following.

### 3.2 On-Surface Synthesis of 4-PA on Ag(110)

**Figure 2:**
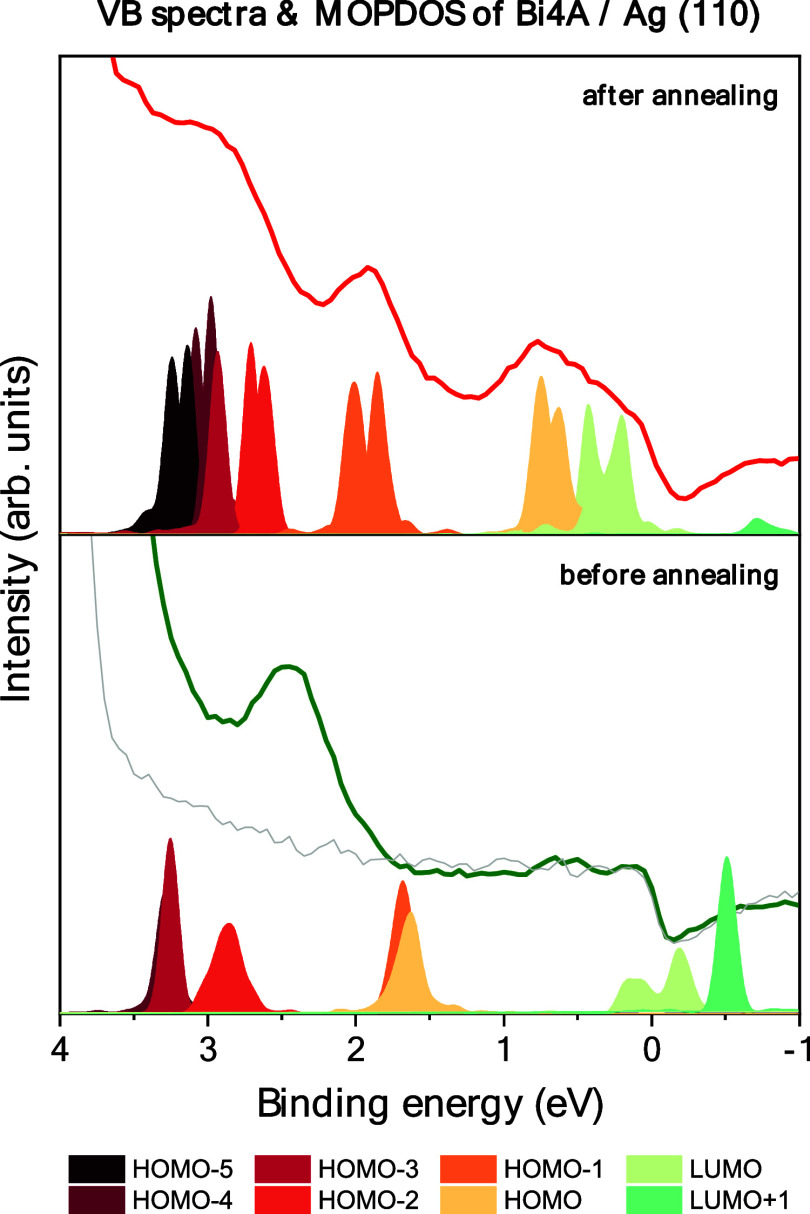
Valence band spectra (lines) and calculated molecular projected density of states (MOPDOS) (filled areas) of a monolayer deposition of Bi4A (bottom) and annealed Bi4A (= 4-PA) (top) on Ag(110). Excitation energy: He I (= 40.8 eV), polar angle: 30°. The gray line in the bottom panel depicts the VB spectra of the clean Ag(110) surface.

Photoemission can be used to obtain valuable information about chemical interactions on surfaces and on-surface reactions. In Figure 2 we show UPS valence band spectra before and after annealing of a 0.4 nm Bi4A film on Ag(110) together with the computed molecular orbital projected density of states (MOPDOS) for Bi4A and 4-PA on the energetically favored adsorption sites. Similar to Bi4A on Cu(111), one can assume that an annealing step is necessary to initiate the cyclodehydrogenation reaction from Bi4A to 4-PA.

In Figure 2, we observe distinct changes of the spectral shape after annealing. Whereas adsorbate related intensity before annealing is found at binding energies higher than 1.8 eV only, additional low binding energy features appear after annealing of the Bi4A monolayer. The MOPDOS in Figure 2 confirms that features with binding energies <1.5 eV are not expected for Bi4A. The difference in the binding energy of the Bi4A HOMO between experiment and computations might be ascribed to different intensity contributions due to incompletely ordered Bi4A films (see below) and/or additional contributions due to the presence in a second layer Bi4A. Most important, the spectrum of the monolayer Bi4A after heating shows excellent consistency with the MOPDOS computations of 4-PA on Ag(110). The dominant features below 1 eV binding energy can be attributed to the highest occupied molecular orbital (HOMO) and the (former) lowest unoccupied molecular orbital (LUMO) of 4-PA. The MOPDOS computations show that a charge transfer from the substrate to the newly formed 4-PA molecule takes place, which leads to the occupation of the 4-PA LUMO. The transferred charge was quantified by performing a Bader charge analysis.^66−69^ For Bi4a/Ag(110) we calculated a charge transfer of 0.21 e and for 4-PA/Ag(110) 1.12 e from the substrate into the molecule. A further indication for the on-surface reaction from Bi4A to 4-PA upon annealing is the development of the work function extracted from UPS data (Figure S4) which is very similar to Bi4A on Cu(111).^37^

**Figure 3:**
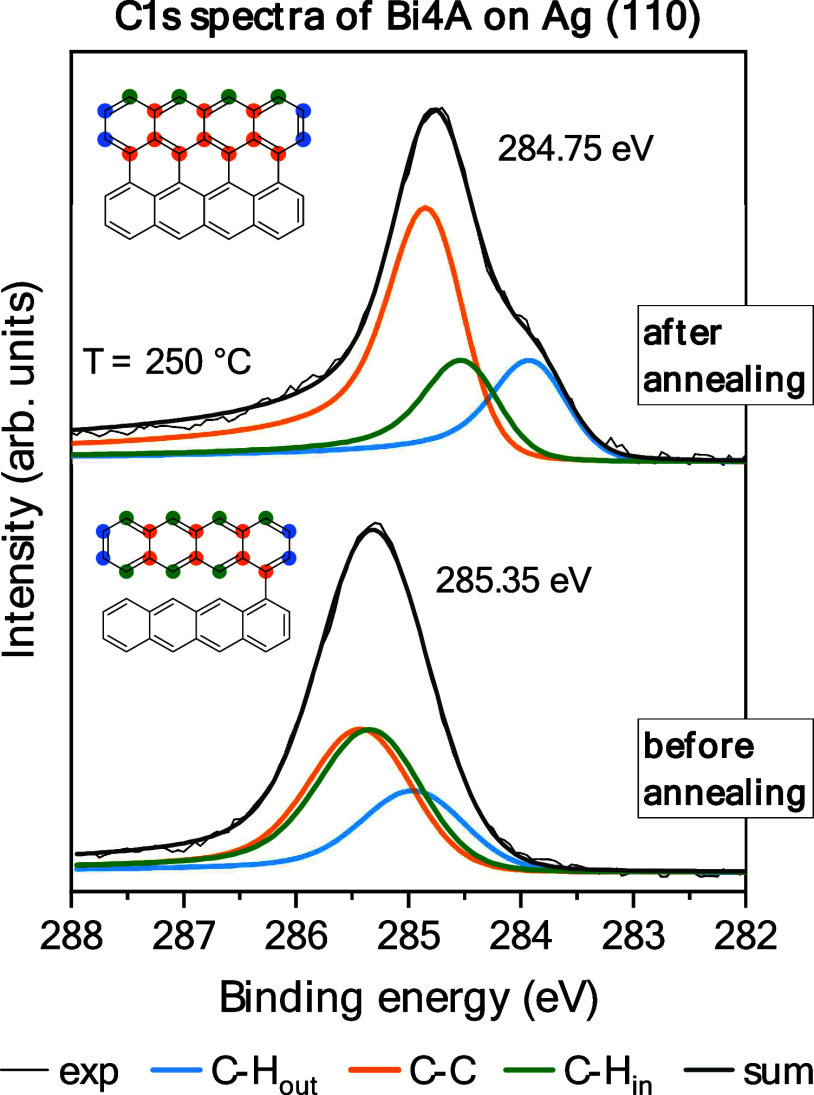
C 1s spectra of a monolayer Bi4A before and after annealing to 250 °C, fitted with three different components. The three components can be attributed to the carbon atoms labeled by different colors.

Core-level photoelectron spectroscopy is well suited to trace on-surface reactions. C 1s core-level spectra of a Bi4A monolayer on Ag(110) before and after annealing are shown in Figure 3. C 1s spectra of larger nanographenes and graphene nanoribbons can be essentially described by two components, assigned to C–C (higher binding energy) and C–H carbon species (lower binding energy).^21^ For acenes and *peri*-acenes, the situation becomes more complicated, as some of the C–H species appear at higher binding energies, overlapping even with C–C.^70−72^ Therefore, the interpretation of the C 1s spectra was facilitated by computed NPA charges that show distinct differences for C–H atoms (Table S1). Moreover, the charge distribution for monolayer coverages is influenced by the metal surface.^72^ In Figure 3, we use a peak fitting model with three components to describe the C 1s spectra qualitatively. An asymmetric Doniach-Sunjic profile had to be applied to adequately describe the increasing tailing to higher energies, which indicates a strong coupling to the metal substrate. The asymmetry factor increases from 0.09 (before annealing) to 0.19 (after annealing), suggesting a stronger coupling to the substrate after annealing. This is in good agreement with the charge transfer evident in the occupation of the former LUMO demonstrated above. All peak fit parameters are summarized in Table S2 in the Supporting Information. The observed changes of the peak shape upon annealing in Figure 3 is qualitatively very similar to Bi4A on Cu(111).^37^ As a consequence of the chemical reaction, a shift to lower binding energy occurs and a decrease of the relative intensity of the low binding energy component is observed. The lower intensity of the low binding energy component compared to Bi4A on Cu(111)^37^ may hint to a more complete surface reaction to 4-PA.

**Figure 4:**
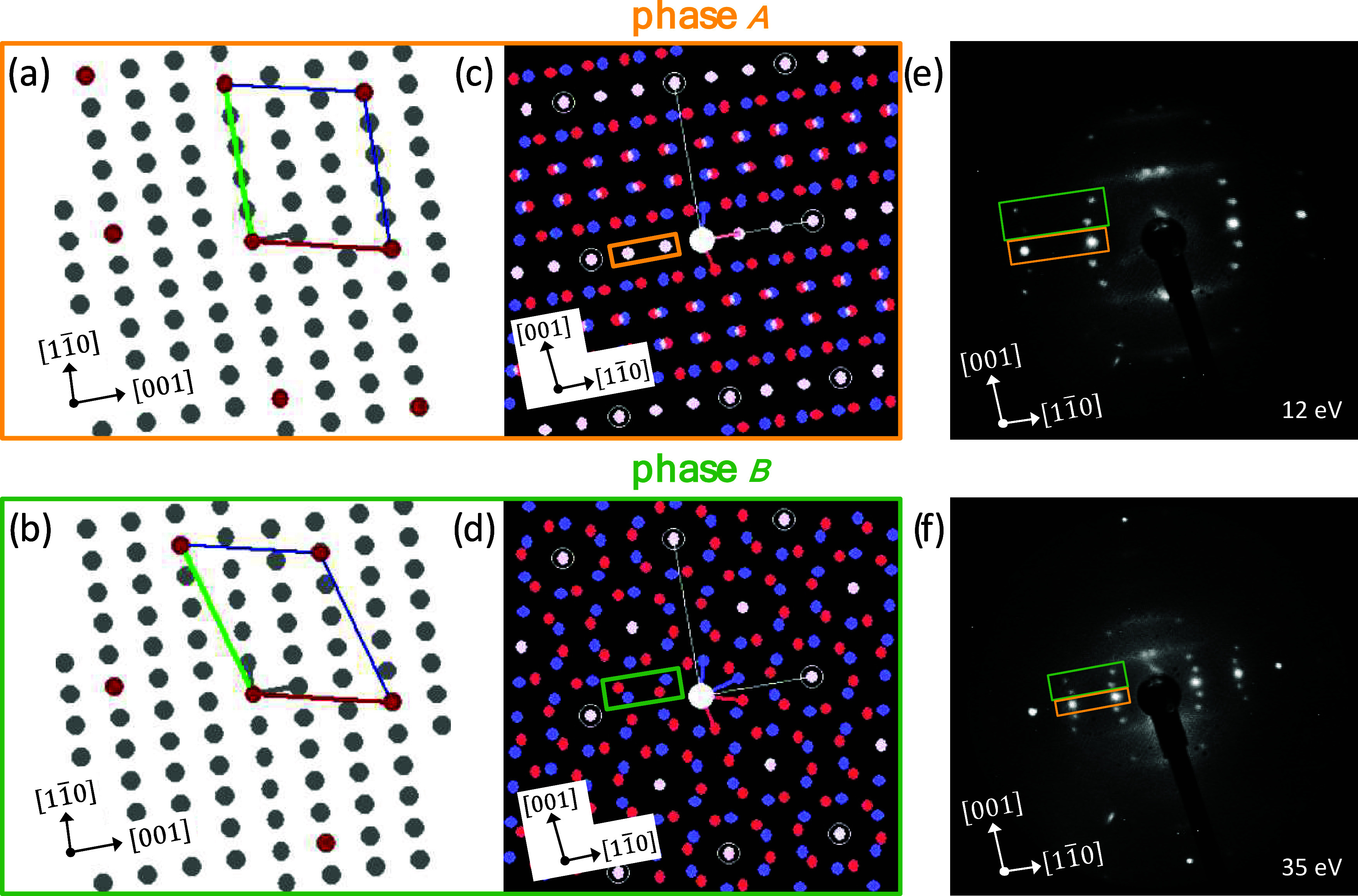
Simulated and experimental LEED images of phase *A* (yellow frame) and phase *B* (green frame) of an annealed Bi4A monolayer deposition on Ag(110): Simulated lattice with a unit cell defined by *r*_1_ = 1.25 nm, *r*_2_ = 1.44 nm, α = 103.2° for phase *A* (a) and *r*_1_ = 1.25 nm, *r*_2_ = 1.49 nm, α = 119.1° for phase *B* (b), simulated diffraction pattern for phase *A* (c) and *B* (d), experimental LEED pattern at a beam energy of 12 eV (e) and 35 eV (f).

In the case of an almost complete on-surface reaction, the 4-PA monolayer should be densely packed and of high symmetry. The long-range ordering was studied by LEED (Figure 4). The experimental LEED images (Figure 4e, f) were recorded at beam voltages of 12 and 35 eV at normal incidence of the electron beam. LEED pattern exhibits sharp spots, which is indicative of the formation of a highly ordered superstructure. We can assign two phases to the LEED pattern in Figure 4. Phase *A* (yellow) grows along the [11̅0] direction, while the superstructure of phase *B* (green) has an angle of +15° relative to the [11̅0] direction. With the help of LEEDpat we simulated the unit cells for both domains, denoted phase *A* and phase *B* in Figure 4. The size of the unit cells is almost identical. While phase *A* is defined by the lattice parameters *r*_1_ = 1.25 nm and *r*_2_ = 1.44 nm, phase *B* is defined by *r*_1_ = 1.25 nm and *r*_2_ = 1.49 nm. The angle between *r*_1_ and *r*_2_ of phase *A* is α = 103.2°, while the unit cell of phase *B* has an angle of α = 119.1°. The unit cells derived from both LEED patterns, described in matrix notation by  (phase *A*, yellow frame) and  (phase *B*, green frame), are in good agreement with a 4-PA molecule, which has dimensions of *r*_1_ = 0.99 nm and *r*_2_ = 1.16 nm.^37^

**Figure 5:**
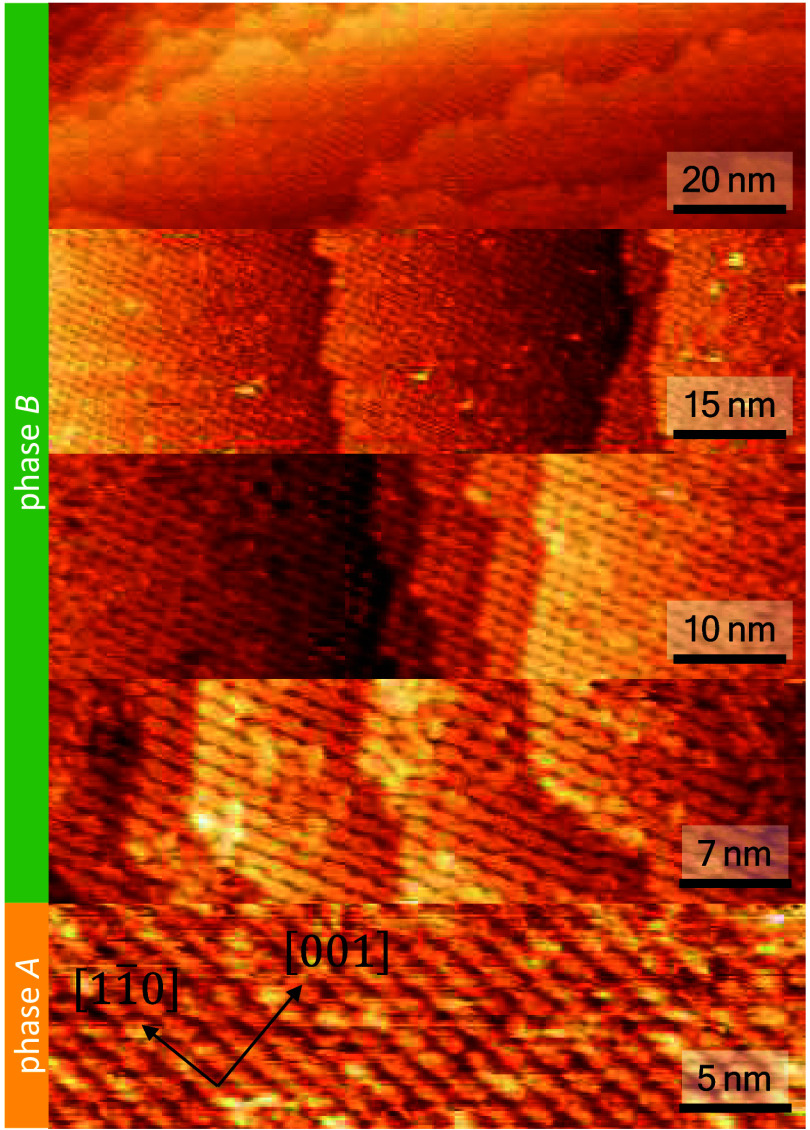
STM images of different scales show high ordering of 4-PA molecules (*U* = −0.3 V, *I* = 300 pA, measured at room temperature). The bottom image shows a homogeneous layer that grows along the [11̅0] direction (phase *A*), while the other four pictures depict that the layer grows rotated by 15 degrees relative to the [11̅0] direction (phase *B*).

The long-range ordering can be further visualized by STM after annealing the monolayer deposition of Bi4A to 250 °C (Figure 5). Different-scale STM images show high ordering along the [11̅0] direction. Ordered structures of single domains can be identified within areas up to at least 200 nm^2^. The bottom image in Figure 5 (scalebar of 5 nm) depicts phase *A*, while the other four pictures show phase *B*, with an angle of +15° relative to the [11̅0] direction. All images were measured on the same sample. It should also be mentioned that the middle images were measured at different positions on the same sample section of 2 × 2 mm^2^. The upper and lower images were measured at different positions on a different sample section. This allows for the tentative conclusion that the ordered areas of phase *A* and *B* could be up to 2 mm in size.

**Figure 6:**
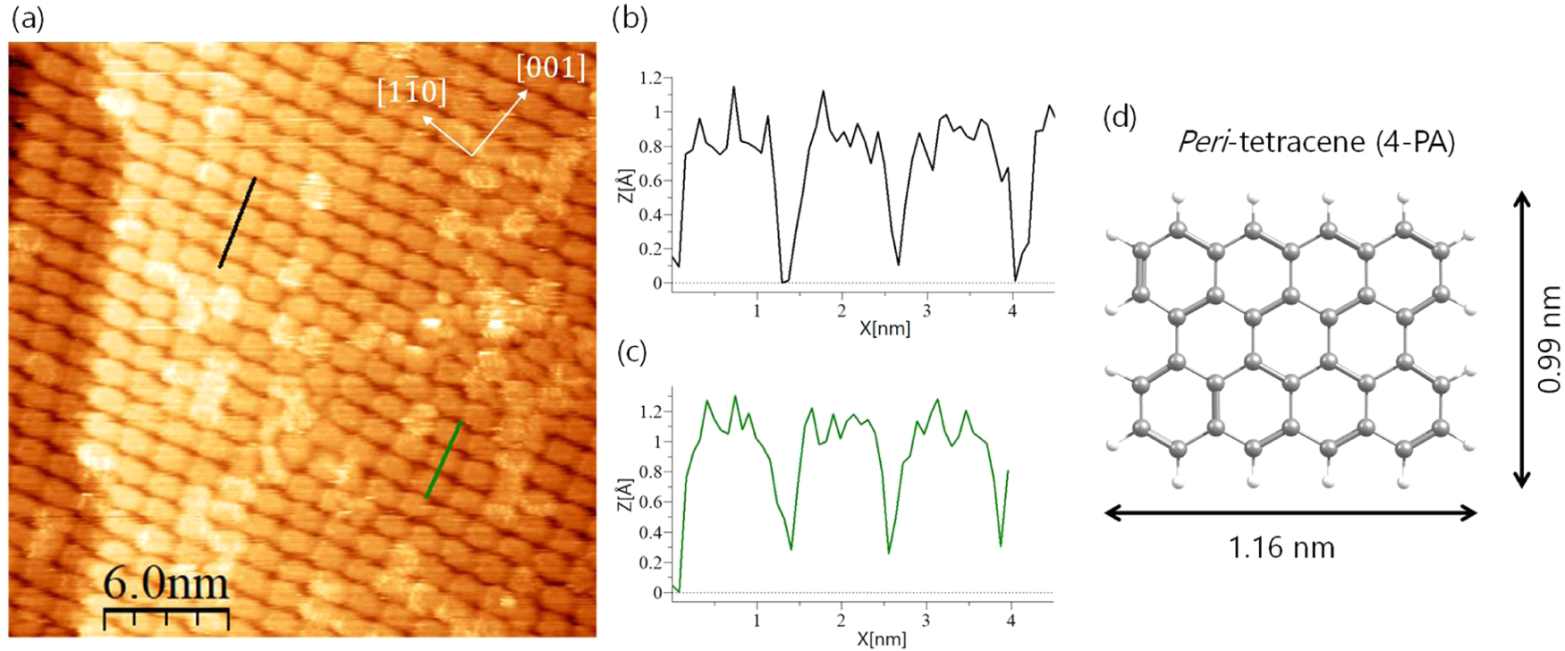
Small-scale STM image (*U* = +0.3 V, *I* = 300 pA, measured at room temperature) with high ordering of a monolayer 4-PA in phase *B* (a). Line profiles shown in (b) and (c) were taken along the black and green line in (a), respectively. The periodicity of about 1.05 nm corresponds well with the short axis of the 4-PA molecule (d).

The detailed arrangement of the molecules is analyzed in Figure 6 by means of a small-scale STM image of a well-ordered region. The line profiles, drawn in the direction of the shorter molecular axis (black and green lines in Figure 6a), have a periodicity of about 1.05 nm and match well with the dimensions of a 4-PA molecule (Figure 6d). It is noteworthy that the 4-PA molecules establish a uniform layer extending beyond the step edge of the substrate. Even on small plateaus and across multiple step edges the periodicity is maintained (see also Figure S5). Thus, this figure illustrates the high quality of homogeneous 4-PA films on Ag(110).

## 4 Conclusions

The present study demonstrates that it is possible to prepare highly ordered large domains of *peri*-tetracene, 4-PA, obtained from 1,1’-bitetracene, Bi4A, by on-surface reaction. The choice of substrate is of enormous importance. While the formation of small-size ordered or disordered 4-PA layers are observed on Cu(111) and Cu(110), respectively, significantly enhanced homogeneity is achieved on Ag(110). These findings are corroborated by DFTB calculations, which reveal variations in adsorption energies for Bi4A and 4-PA across different adsorption sites and crystallographic directions on Cu(111), Cu(110), and Ag(110) surfaces. Experimental investigations, including UPS, XPS, LEED, and STM measurements, provide evidence of homogeneous large-area 4-PA layers on Ag(110). Notably, the valence band spectrum of annealed Bi4A (= 4-PA) exhibits a novel peak between 0.3 and 0.8 eV, corresponding to the HOMO and former LUMO as predicted by the molecular-orbital projected DOS analysis based on our hybrid functional DFT calculations. The LUMO is filled by charge transfer from the Ag substrate to the molecule. Two different phases were observed by LEED and STM, which differ slightly in their molecular alignment with respect to the metal surface: Phase *A* aligns exactly along the [11̅0] direction of the substrate, while phase *B* is rotated by 15°. Despite this difference in orientation, the unit cells of the two phases are nearly identical. STM images further confirm the high degree of ordering within the molecular layer and demonstrate the coexistence of both phases within a single experiment, albeit at different regions of the crystal. This observation indicates that a single phase can dominate across large areas of the surface. Additionally, it was observed that step edges on the Ag(110) substrate do not disrupt the homogeneity of the 4-PA layer, as the molecular layer continues to grow seamlessly across multiple step edges. These findings underscore the suitability of the Ag(110) surface as a substrate for the formation of highly ordered 4-PA layers using Bi4A as a precursor molecule.

## Supporting Information Available

Additional STM images; description of main adsorption sites on a [111] and [110] single crystal; additional work function measurements; natural charges of 4-PA and Bi4A, and corresponding peak fit data (PDF)
