# Supplementary material for: Highly Ordered Single Domain Peri-Tetracene Monolayers on Ag(110)
Source: J Phys Chem C Nanomater Interfaces. 2025 Apr 15;129(17):8447–54. doi: 10.1021/acs.jpcc.5c01482 (PMC12051201; doi:10.1021/acs.jpcc.5c01482)
Supplement: Supplementary file 1 — jp5c01482_si_001.pdf [file jp5c01482_si_001.pdf]

Supporting Information

Highly Ordered Single Domain *Peri*-Tetracene Monolayers on Ag (110)

Maren Zirwick,[a, b] Nina Kainbacher,[c] John B. Bauer,[b] Marie S. Wagner,[a, b] Peter Puschnig,[c] Thomas Chassé,[a] Holger F. Bettinger,[b, d, *] Heiko Peisert[a, *]

[a]*Institute of Physical and Theoretical Chemistry, University of Tübingen, 72076 Tübingen, Germany*

[b]*Institute of Organic Chemistry, University of Tübingen, 72076 Tübingen, Germany*

[c]*Institute of Physics, NAWI Graz, University of Graz, 8010 Graz, Austria*

[d]*Center for Light-Matter Interaction, Sensors & Analytics (LISA+) at the University of Tübingen, 72076 Tübingen, Germany*

*Corresponding authors: heiko.peisert@uni-tuebingen.de, holger.bettinger@uni-tuebingen.de

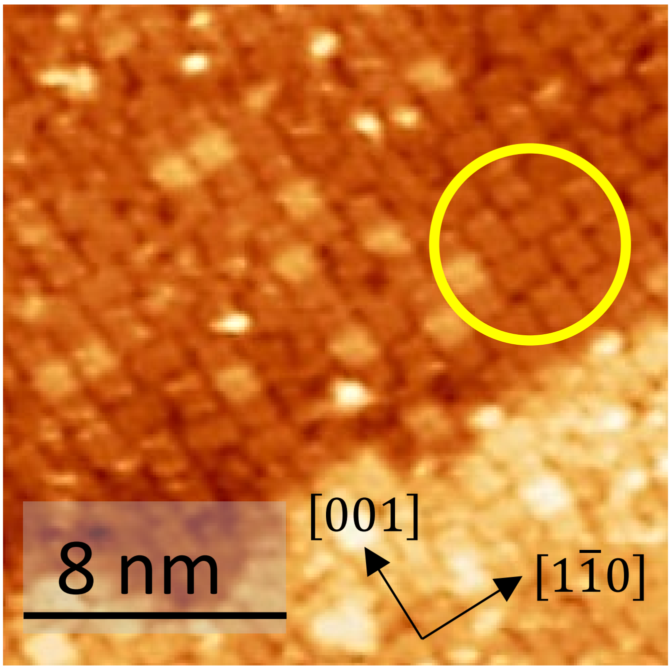

Figure S1: STM image of a monolayer deposition of Bi4A / Cu (110) annealed to 250 °C (U = - 0.1 V, I = 680 pA, measured at room temperature). The yellow circle highlights four peri-tetracene molecules lying in [1$\bar{1}$0] *and* [001] *direction, respectively.*

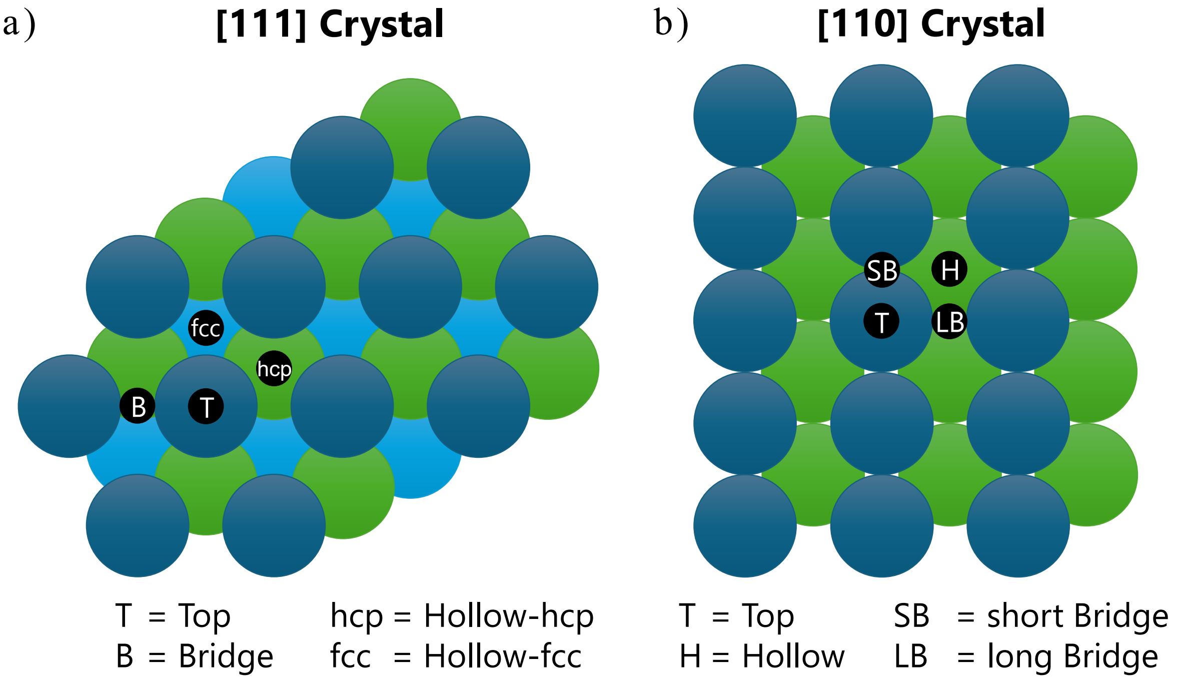

ك

Figure S2: The four main adsorption sites on a [111] (a) and [110] crystal (b). The top layer is marked in dark blue, the second layer in green and the third layer in light blue.

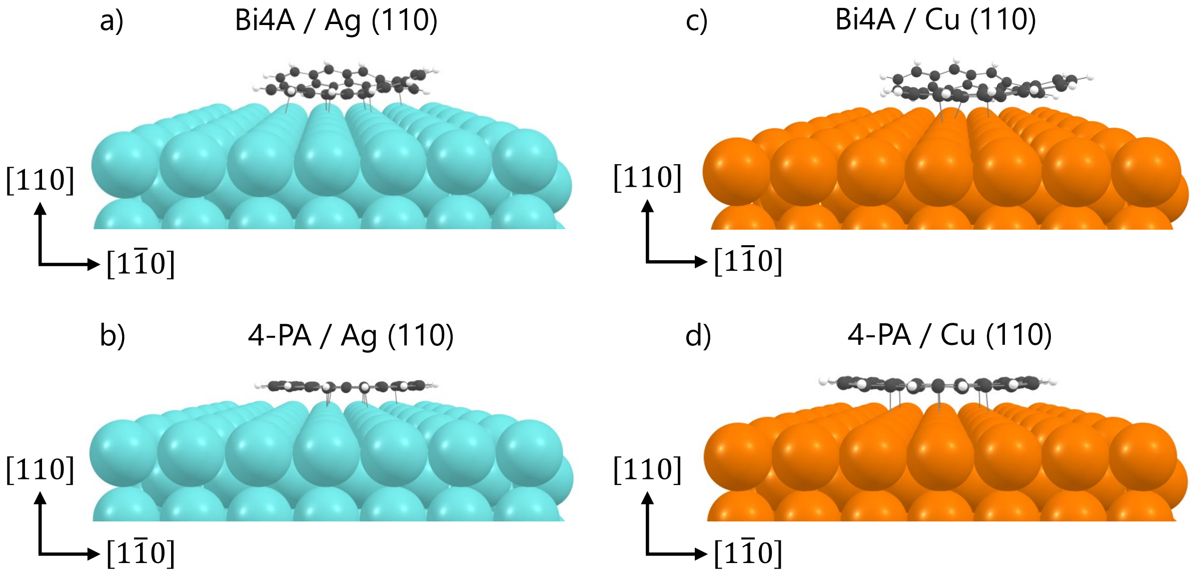

Figure S3: Sideview of the preferred adsorption site of Bi4A and 4-PA on Ag (110) (a, b) along the [1$\bar{1}$0]*-direction and adsorption site of Bi4A and 4-PA on Cu (110) along the* [1$\bar{1}$0]*-direction (c, d) as computed using the DFTB method.*

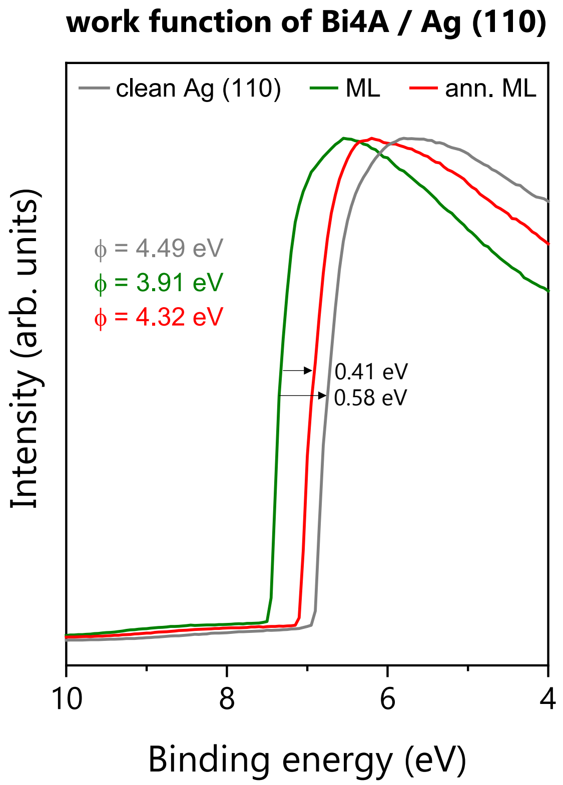

Figure S4: Work functions of a monolayer deposition of Bi4A before and after annealing and of the clean Ag (110) surface obtained at normal emission and an excitation energy of He I, 21.2 eV.

The work function of the monolayer Bi4A / Ag (110) is reduced by 0.58 eV compared to the clean Ag (110) crystal surface.

Table S1: Natural charges of the inequivalent carbon atoms in Bi4A (left) and 4-PA (right) according to the natural population analysis (B3LYP/def2-TZVP).

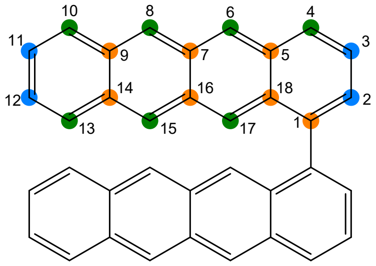

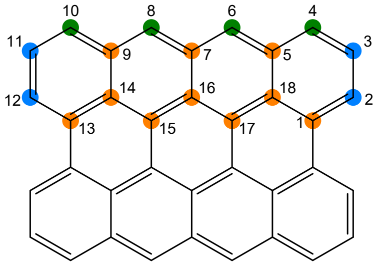

Atom	NPA charge	Atom	NPA charge
C 1	-0,01261	C 10	-0,17708
C 2	-0,19574	C 11	-0,20751
C 3	-0,20557	C 12	-0,20872
C 4	-0,17740	C 13	-0,17532
C 5	-0,05502	C 14	-0,05782
C 6	-0,14957	C 15	-0,14596
C 7	-0,05531	C 16	-0,05501
C 8	-0,15055	C 17	-0,15100
C 9	-0,05613	C 18	-0,05175

Atom	NPA charge	Atom	NPA charge
C 1	-0,02369	C 10	-0,17577
C 2	-0,18062	C 11	-0,20118
C 3	-0,20118	C 12	-0,18062
C 4	-0,17577	C 13	-0,02368
C 5	-0,05212	C 14	-0,03534
C 6	-0,14974	C 15	+0,05156
C 7	-0,05364	C 16	-0,05622
C 8	-0,14973	C 17	+0,05156
C 9	-0,05213	C 18	-0,03534

Table S2: Peak fit data of the C 1s core levels of the evaporated and annealed ML.

**Peak name**	**GP [eV]**	**LP [eV]**	**Position [eV]**	**FWHM [eV]**	**Rel. area [%]**	**Asymmetry**
1,1'-bitetracene						
**C-C**	0.97	0.1	285.38	1.102	38.9	0.09
**C-H$_{in}$**	0.97	0.1	285.30	1.102	38.8	0.09
**C-H$_{out}$**	0.97	0.1	284.91	1.102	22.3	0.09
ann. 1,1'-bitetracene						
**C-C**	0.66	0.1	284.77	0.843	55.2	0.19
**C-H$_{in}$**	0.66	0.1	284.46	0.842	22.3	0.19
**C-H$_{out}$**	0.66	0.1	283.85	0.842	22.6	0.19

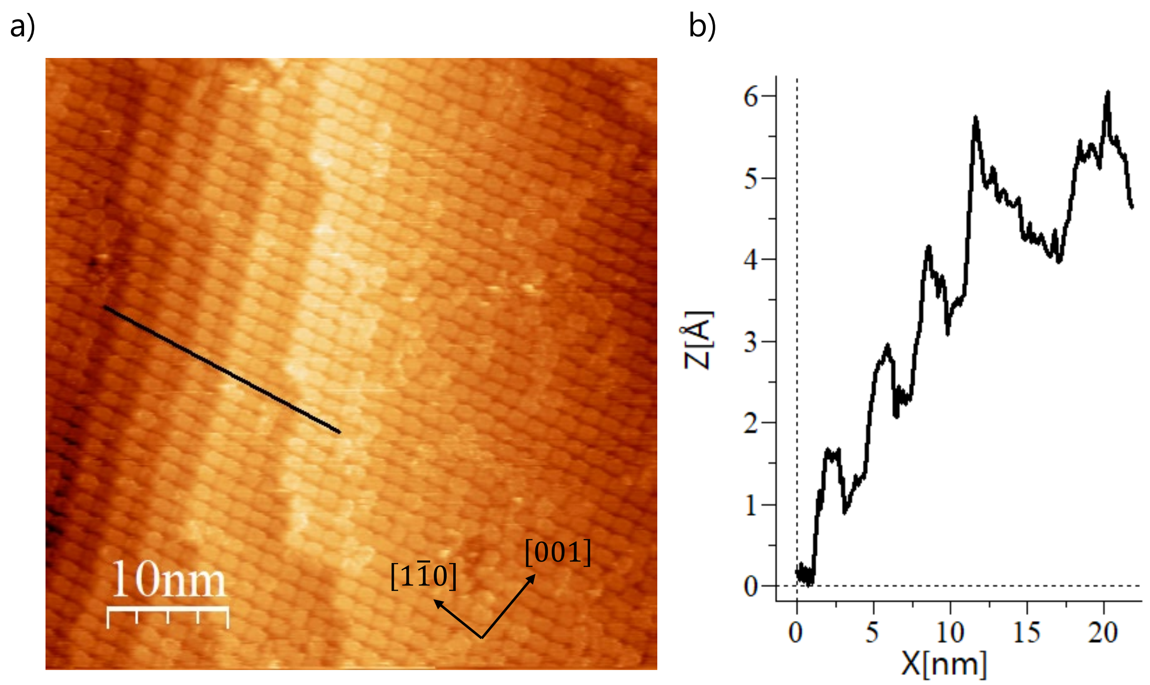

Figure S5: STM image of 4-PA monolayer on Ag (110) (U = + 0.3 V, I = 300 pA, measured at room temperature) (a) and a topological profile over 5 step edges (b). The layer continues to grow homogeneously beyond the step edges.
